# Anti-Inflammatory Activity of the Essential Oil Citral in Experimental Infection with* Staphylococcus aureus* in a Model Air Pouch

**DOI:** 10.1155/2017/2505610

**Published:** 2017-02-21

**Authors:** Hellen Braga Martins, Nathan das Neves Selis, Clarissa Leal Silva e Souza, Flávia S. Nascimento, Suzi Pacheco de Carvalho, Lorena D'Oliveira Gusmão, Jannine dos Santos Nascimento, Anne Karoline Pereira Brito, Samira Itana de Souza, Marcio Vasconcelos de Oliveira, Jorge Timenetsky, Regiane Yatsuda, Ana Paula T. Uetanabaro, Lucas M. Marques

**Affiliations:** ^1^State University of Santa Cruz (UESC), Campus Soane Nazaré de Andrade, Ilhéus, BA, Brazil; ^2^Multidisciplinary Institute of Health, Federal University of Bahia (UFBA), Vitória da Conquista, BA, Brazil; ^3^Department of Microbiology, Institute of Biomedical Science, University of São Paulo (USP), São Paulo, SP, Brazil

## Abstract

This study proposes to implement an alternative and effective strategy for local treatment of disease provoked by* S. aureus*. For the analysis of possible anti-inflammatory activity of essential oil, after establishing an air pouch model, 48 male mice of Balb/c were treated, infected, and euthanized at 4 and 8 h. Thus, the total and differential white blood cells were counted in the animal's blood, and cytokines IL-1*β*, IL-6, and TNF-*α* were titrated using ELISA in the air pouch lavage. Moreover, TNF-*α*, IL-1*β*, and IL-6 gene expression was analyzed through an RT-qPCR array, and* S. aureus* was quantified using qPCR. Our results, *p* < 0.05, showed that EOC reduced the quantity of microorganisms. The group of mice treated with essential oil citral showed a significant decrease in TNF-*α* levels in tests demonstrating anti-inflammatory activity. There is no data about the mutual influence of the air pouch model, essential oil citral, and* S. aureus*. Thus, considering the interaction of these variables and the anti-inflammatory activity of the essential oil citral, we demonstrated, by alternative local treatment, a new antimicrobial agent that is not an antibiotic.

## 1. Introduction

*Staphylococcus aureus* is among the most pathogenic species of the staphylococci group, being responsible for causing endemic and epidemic infections, and can be acquired in hospitals or in the community, resulting in a high rate of morbidity and mortality worldwide [1]. Being an opportunistic pathogen, 20 to 30% of* S. aureus* is found on the skin and airways of the humans, being isolated from the skin environment and skin microbiota, respiratory tract, and other places [2].

This species may cause a host of illnesses ranging from mild skin diseases to potentially lethal diseases, including respiratory infections, bacteremia, septic arthritis, endocarditis, osteomyelitis, diarrhea, deep abscesses, and toxic shock syndrome. It can also lead to the development of diseases that compromise immunity, thus predisposing both healthy and immunocompromised individuals to the emergence of infections [3].

To eliminate this pathogen therapeutic measures play a critical role, since the host defense against* S. aureus* relies mainly on innate immunological mechanisms of the immune system. Initially, administering a broad-spectrum of antibiotics is recommended. However, because of antibiotic resistance and the increasing prevalence of MRSA (methicillin-resistant* Staphylococcus aureus*), vancomycin has been increasingly used as an experimental measure [4, 5].

A promising alternative treatment for infections that should be considered is essential oils (EO), so called because of their composition based on lipophilic substances [6]. Essential oils are volatile secondary metabolites, which act through the cell wall and cell membrane, which may affect bacterial structures because of their cytotoxic and therapeutic properties, and protection against oxidation processes and deterioration caused by microorganisms [7, 8].

The essential oil citral (EOC) is composed of major constituents of monoterpenes (isomeric mixture of geranial and neral) and myrcene being found in a wide variety of plants, such as the lemon balm* (Melissa officinalis)* in lemon grass or lemongrass* (Cymbopogon citratus)* and verbena* (Verbena officinalis)* [9].

This oil has antibacterial and antifungal activity, analgesic and antispasmodic effects on uterine and intestinal tissue, antiparasitic action, and activity on the central nervous system, as well as being an anxiolytic and sedative, and these therapeutic properties of the compounds are explained by the presence of monoterpenes and myrcene. In addition to being antibacterial, EOC acts against gram-positive and gram-negative bacteria, and prolonged exposure to its use has not encountered resistance, thus justifying its use [10, 11].

The high mortality rate related to staphylococcal infections [12], the increase in* S. aureus* isolates resistant to antibiotics [13], and frequent and prolonged exposure to antimicrobial agents hinder the treatment of infections [14]. Therefore, alternative strategies to prevent or treat infections caused by* S. aureus*, sometimes minimizing or supplementing use of antibiotics, are necessary. Given the above background, with the changes in the epidemiology of infections, increased prevalence of resistance and significantly higher rates of morbidity and mortality, it is necessary to develop new treatment strategies for infections triggered by* S. aureus* [15]. Thus, we aim to analyze the anti-inflammatory activity of essential oil citral in experimental inoculation of* S. aureus* in an air pouch model, to provide solutions in local infections, promoting alternative therapeutic approaches to antibiotics and linking to advances in biotechnology.

## 2. Materials and Methods

This study was performed after approval by the Ethics Committee on Animal Use (CEUA) of the Multidisciplinary Institute of Health Campus Teixeira (IMS/CAT) of the Federal University of Bahia, with protocol number 021/2014.

### 2.1. Microorganism

For inflammatory induction, the reference strain of* Staphylococcus aureus* ATCC® 33591™ was used. This microorganism is resistant to the antibiotic methicillin (MRSA), positive mecA, nuc positive and PVL negative (according to data from The Global Bioresource Center). Brain Heart Infusion (BHI) and mannitol salt agar (MSA) were used for activation, reactivation, and cultivation of the strain of* S. aureus*, stored in a freezer at −70°C for subsequent inoculation in animals. For reactivation of virulence factors of the strain, concentrated inoculum of the plated bacteria was prepared with sterile saline, and an amount of 20 *μ*L was applied on the ear of male BALB/c mice anesthetized intraperitoneally with 100 uL containing ketamine hydrochloride (40 mg/Kg) and xylazine (10 mg/Kg). After 24 h, it was euthanized by increasing anesthesia followed by exsanguination, and the node of the throat, the same ear side used for inoculation, was removed with the aid of sterile surgical instruments and transferred to a falcon tube containing 1 mL of medium RPMI®. At the sediment, the sample was poured in a Petri dish to which 1 mL of medium RPMI was added further to be macerated. From the supernatant, 30 *μ*L was transferred to a plate with MSA medium for bacterial growth and maintained in a bacteriological oven at 37°C for 24 h.

### 2.2. Essential Oil Citral

In the experiments we used essential oil citral (EOC) provided commercially by Sigma®, and the dosage (40 mg/Kg) used was recommended according to the literature review [16–18], in which less answer variations were presented. For the test run, the citral was diluted in Tween 1%.

### 2.3. Animal

Forty-eight male BALB/c mice, free of specified pathogens (SPF), were used at 8 weeks of age and were weighing on average 30 g. The animals were kept in an environment with controlled and uniform conditions, with temperature of 21 ± 2°C and humidity of 50 ± 2%, and subjected to the same procedures within each group. For the challenge, groups of twelve animals were made, six animals euthanized at the time of 4 h and six at the time of 8 h, as follows:

  C: uninfected mice, but with the administration of Tween 1%, euthanized after 4 and 8 hours
  CT: uninfected mice, but with the administration of the essential oil citral, euthanized after 4 and 8 hours
  S: mice infected with* S. aureus* and euthanized after 4 and 8 hours
  S + CT: mice infected with* S. aureus* and treated with essential oil citral and euthanized at 4 and 8 hours

The mice of group C were anesthetized with a 100 mL solution containing ketamine hydrochloride (40 mg/Kg) and xylazine (10 mg/Kg) by intraperitoneal injection, for developing a sterile compartment called an air pouch; 3 mL of sterile air was injected into the back of the animal, followed by 100 *μ*L Tween 1% injected into the air pouch. The mice of group CT were inoculated intraperitoneally at first with 100 mL of essential oil citral and anesthetized and then for developing the air pouch 3 mL of sterile air was injected into the back of the animal. The mice of group S were anesthetized for developing an air pouch, and 3 mL of sterile air was injected into the back of the animal. Next, the mice were challenged with an injection of 100 *μ*L of ATCC 33591 strain into the air pouch. The inoculum was carried out by direct suspension, drawing from 3 to 5 colonies from the plates with mannitol salt medium, using a bacteriological loop. After this step, following protocol of CLSI (Clinical & Laboratory Standards Institute), one tube aliquot was removed and placed in quartz cuvettes for reading a spectrophotometer to obtain the inoculum, following these parameters: 0.135 Abs (660 nm), equivalent to 1 × 10^8^ CFU/mL (colony forming units/mL). The mice of S + CT group were inoculated intraperitoneally on the first time with the essential oil citral. After 30 minutes, they were anesthetized for developing an air pouch, by injecting 3 mL of sterile air into the back of the animal. Later, they were challenged with an injection of 100 *μ*L of ATCC 33591 strain, with inoculum carried out as stated in the S group. The animals were euthanized by increasing the anesthetic and exsanguination after 4 or 8 hours, according to experimental guidelines. Later, the same were subjected to a surgical procedure to remove the blood and wash the air pouch and the skin around the air pouch.

### 2.4. Obtaining Blood and Washing Skin and Air Pouch

Immediately after euthanasia, blood samples from animals were collected in tubes with and without EDTA for the total and differential leukocyte count. After trichotomy, the air pouch lavage was obtained by injecting 3 mL of sterile saline solution, proceeding with massage for homogenization. The fluid obtained was stored for analysis of the cytokines.

### 2.5. Leukocyte Count (Total and Differential)

To quantify the total leukocytes, 20 mL of blood (collected in EDTA) was mixed with 400 *μ*L of fluid diluter (TURK). After 20 min., the sample was transferred to a Neubauer chamber and then the optical microscope to perform the leukocyte count in a 40x magnification. The rating differential was performed with a 100-leukocyte count, stained with Quick Kit Dye (Panotic) at a 100x magnification, distinguishing them according to their type (neutrophils, monocytes, and lymphocytes). Two methods were used for counting the longitudinal and zigzag pattern. The absolute values for each leukocyte were calculated based on the total leukocyte count and percentage values found in the differential count.

### 2.6. Quantification of* S. aureus*

The genomic DNA of the samples was extracted according to the protocol for an Invitrogen Purelink™ Genomic DNA Kit (Invitrogen, São Paulo, SP, Brazil). Real-time PCR was performed in duplicate, using the Applied Biosystems™ platform. The technique was carried out by using TaqMan probes, using an amplification based protocol with a final volume of 25 *μ*L of reaction: 12.5 *μ*L of Master Mix (Applied Biosystems), 1.12 *μ*L of primer LTnucF (AAATTACATAAAGAACCTGCGACA), 1.12 *μ*L of primer LTnucR (GAATGTC ATTGGTTGACCTTTGTA) (20 pmoL), which are single copy genes, 0.75 *μ*L of probe (10 mM), 7.0 *μ*L of water, and 2.5 *μ*L of DNA. The parameters for amplification are 95°C-10 min, followed by 30 cycles (95°C, 15 seconds; 59°C, 1 min). Quantitation was performed using an absolute quantization technique, based on a predetermined standard curve ranging 10^7^–10 microorganisms/*μ*L. A new curve was added to each reaction, and the following parameters were established: *r*^2^ ≥ 0.950 and efficiency between 95 and 105%.

### 2.7. Dosage of Cytokines

The dosage of TNF-*α*, IL-1*β*, and IL-6 cytokines was determined in the washed air pouch by ELISA (enzyme-linked immunosorbent assay) capture, according to the kit manufacturer guidelines (eBioscience, San Diego, CA, USA), in 96-well polystyrene plates, which confer high absorption. The reading was performed using a microplate reader Vivid Vision instrument with a wavelength of 450 nm.

### 2.8. Gene Expression

The gene expression of inflammatory markers was assessed by an RT-qPCR array methodology. The mRNA of the air pouch skin samples was extracted by using Trizol, following the protocol supplied by the manufacturer. The obtained cDNA was performed using SuperScript® III Reverse Transcriptase Kit. The test gene expression SYBR® PCR Master Mix (Applied Biosystems) was conducted in custom-plates with the target genes IL-1*β*, IL-6, and TNF-*α*, with endogenous genes glyceraldehyde 3-phosphate dehydrogenase (GAPDH) and *β*-actin, and genes MGDC control (Mouse Genomic DNA Contamination), RTC (Reverse Transcription Control), and PPC (Positive PCR Control). Amplification was performed in thermocycler StepOnePlus™ Software v2.3, with the following parameters for all genes: 95°C for 10 minutes, followed by 40 cycles at 95°C for 15 seconds and 60°C for 1 min.

### 2.9. Immunohistochemistry

After processing and inclusion of samples in paraffin, histological sections with a thickness of 4 mm were used to study the immunohistochemistry. The sections were deparaffinized through a series of “baths” in xylene and alcohol. With the aim of improving the responses, one treatment stage of the blades was performed with citrate buffer. Endogenous peroxidase was blocked using a solution composed of methanol and hydrogen peroxide (30%). Antibodies were used specific for macrophages CD 68 (AbD Serotec), being a marker of inflammatory response in tissue. A biotinylated anti-mouse secondary antibody was used (ImmPRESS™ Universal Reagent, Vector Laboratories). Subsequently, we used the avidin-biotin complex. The color development occurred by adding the chromogen 3,3-diaminobenzidine (DAB, 3,3-diaminobenzidine, Easypath®) on the cuts by the reaction thereof with the avidin-biotin-peroxidase complex. The counterstaining was conducted by Harris hematoxylin bath. Twelve microscopic fields were analyzed with 40x magnification for counting the labeled cells in the ImageJ program.

### 2.10. Statistical Analysis

For analysis of cell count data, cytokines, and gene expression, GraphPad Prism 6.0 was used. Nonparametric tests were used: Kruskal-Wallis (when evaluating more than two groups) and Mann–Whitney (when evaluating two groups). Statistical differences were considered significant at *p* < 0.05 using a 95% confidence interval.

## 3. Results

**Figure 1:**
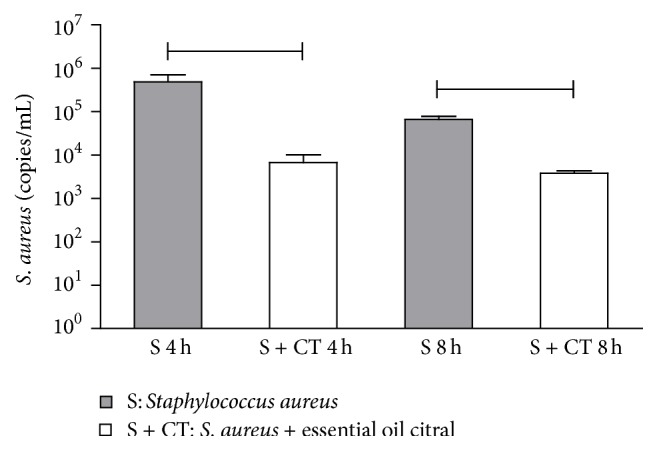
Quantification by qPCR of* S. aureus* (CFU/*μ*L) in air pouch. Groups (*n* = 6):* S. aureus* (S) and* S. aureus* treated with citral (S + CT). 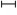
Significant difference using *p* < 0.05, Mann–Whitney test.

By means of qPCR it was possible to quantify DNA and consequently the microbial load of S and S + CT groups in the samples of the washed air pouch being represented by the same colony forming units (CFU)/*μ*L. Thus, in Figure 1, one can observe high probes (>10^4^ and <10^5^ CFU/*μ*L) of* S. aureus*; however, the average of the groups was greater in the group infected at 4 h. Compared to the treated group, this could be an indication that the EOC had a positive effect in reducing the microorganism analyzed.

**Figure 2:**
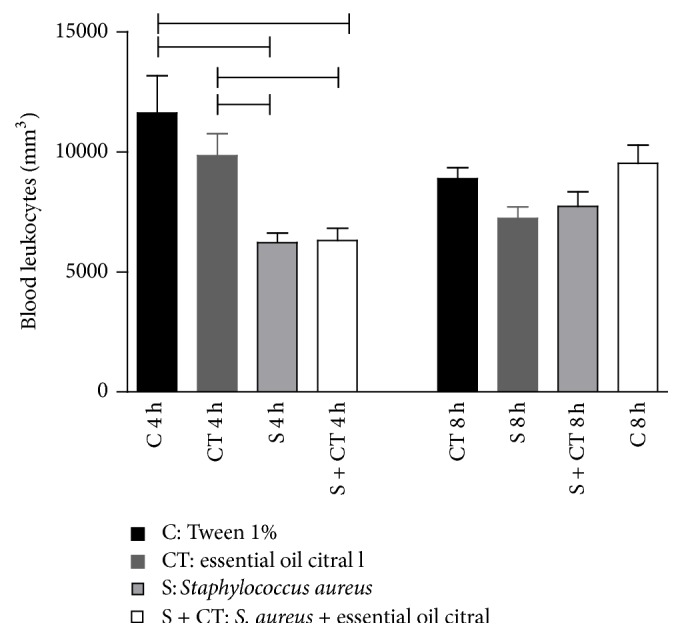
Quantification of total leukocytes in the blood of Balb/c after 4- and 8-hour inoculation. Groups (*n* = 6) inoculated with Tween 1% (C), citral (CT),* S. aureus* (S), and* S. aureus* treated with citral (S + CT), in an experiment lasting 4 and 8 h. 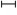
Significant difference using *p* < 0.05, Kruskal-Wallis test.

In order to analyze the air pouch model in mice, leukocyte counts of all the animals were done after euthanasia. The group intraperitoneally inoculated with the EOC induced quantitative alterations in leukocytes in peripheral blood. The evaluation of the total count showed significant differences (*p* < 0.05) at 4 h in the group administered with Tween 1% (C) or EOC (CT) compared to the infected group (S) or infected and treated group (S + CT); see Figure 2. However, there were no differences in the total number of leukocytes in the S to S + CT groups in the two days of analysis.

**Figure 3:**
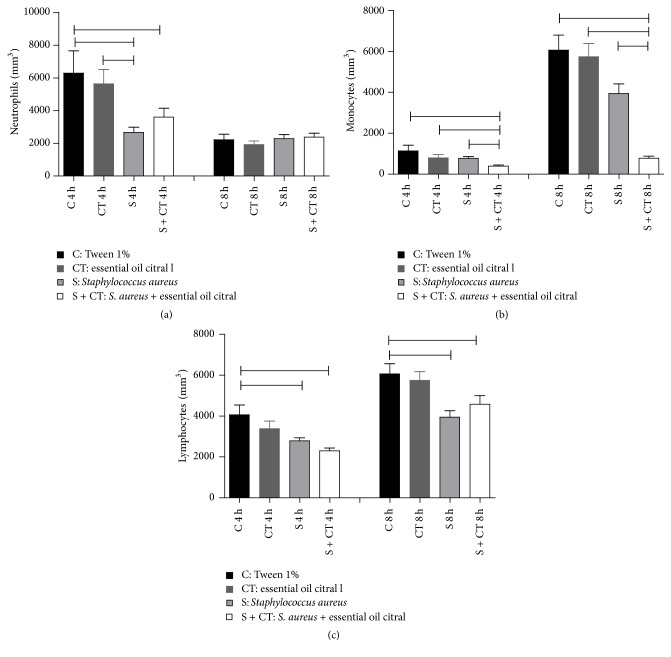
Number of neutrophils (a), monocytes (b), and lymphocytes (c) in the blood of Balb/c mice after 4- and 8-hour inoculation. Groups (*n* = 6) inoculated with Tween 1% (C), citral (CT),* S. aureus* (S), and* S. aureus* treated with citral (S + CT), in an experiment lasting 4 and 8 h. 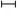
Significant difference using *p* < 0.05, Kruskal-Wallis test.

To evaluate the relative quantities of different types of leukocytes in peripheral blood leukocytes, differential counting was performed. Regarding the number of neutrophils, monocytes, and lymphocytes, there were statistically significant differences (*p* < 0.05) in these hematological parameters between the control and treated groups. Differences were observed in the neutrophil groups C and S; C and S + CT; S and CT. Differences were observed for lymphocytes in groups C and S; C and S + CT in the study time. However, comparing the S group to the S + CT group only in regard to monocytes (Figure 3), significant differences can be seen at 4 h and 8 h of infection.

**Figure 4:**
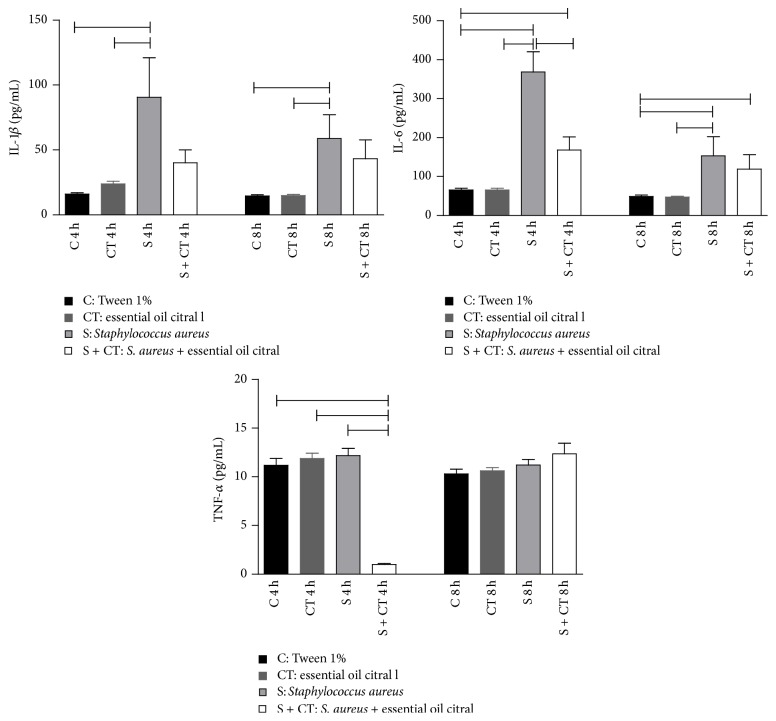
Levels of cytokines IL-6, IL-1*β*, and TNF-*α* in air pouch from Balb/c mice after 4 and 8 hours' inoculation. Groups (*n* = 6) inoculated with Tween 1% (C), citral (CT),* S. aureus* (S) and* S. aureus* treated with citral (S + CT), in an experiment lasting 4 and 8 h. 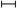
Significant difference using *p* < 0.05, Kruskal-Wallis test.

Among endogenous mediators, cytokines play an important role in the host response, and in the dosage of TNF-*α*, IL-1*β*, and IL-6 markers in the lavage, it was possible to assess inflammatory responses in groups. Thus, in Figure 4, we can notice significant differences for the three cytokines, *p* < 0.05. However, the group of infected mice compared with the infected and treated group with the EOC in the time of 4 h showed a significant decrease in IL-6 and TNF-*α* levels. IL-1*β* levels decreased in groups S and S + CT at 4 h; however, the data was not statistically significant.

**Figure 5:**
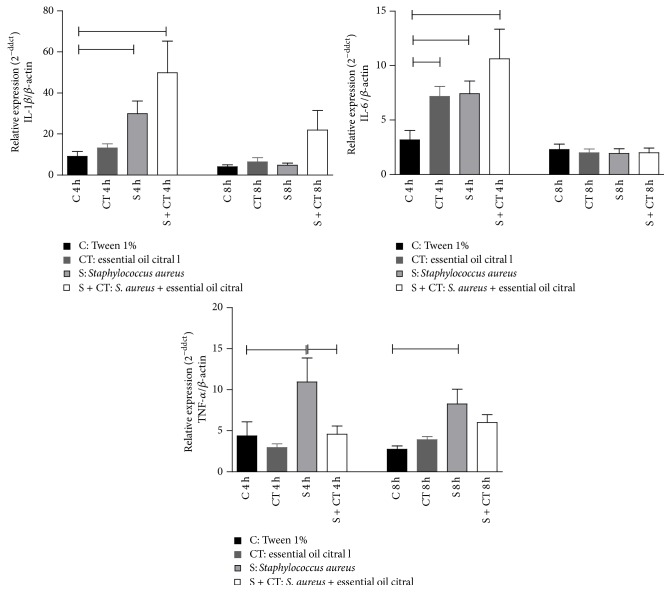
Gene expression of IL-1*β*, IL-6 and TNF-*α* in air pouch from Balb/c mice after 4 and 8 hours' inoculation. Groups (*n* = 6) inoculated with Tween 1% (C), citral (CT),* S. aureus* (S) and* S. aureus* treated with citral (S + CT), in an experiment lasting 4 and 8 h. 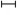
Significant difference using *p* < 0.05, Kruskal-Wallis test.

Analysis of the expression of TNF-*α*, IL-1*β*, and IL-6 genes showed less variation among the different treatments, where statistical significance was observed between the three genes, *p* < 0.05. However, evaluating the inflammatory state induced in the group infected with* S. aureus* against the infected group and treated with EOC, differences can be observed only for the TNF-*α* gene at 4 h, *p* < 0.05 (Figure 5).

**Figure 6:**
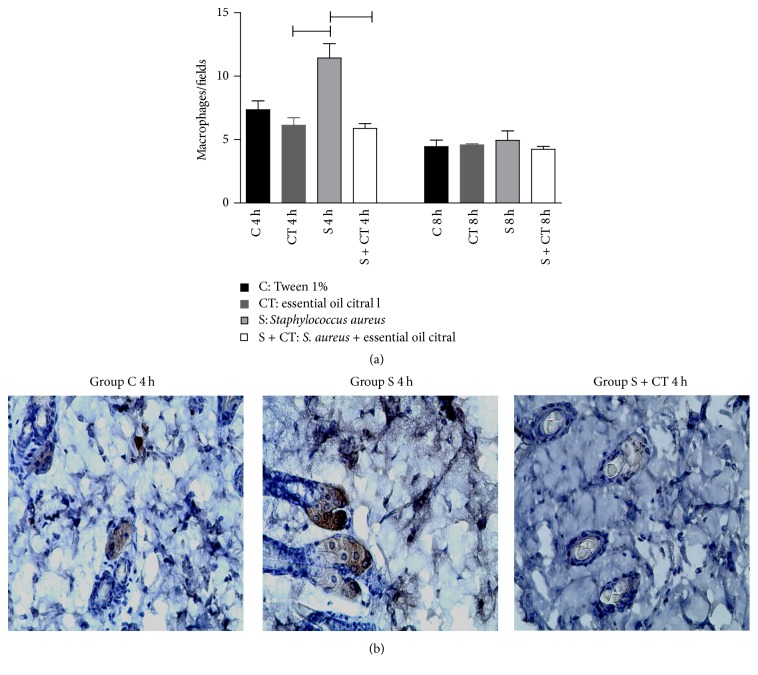
Immunolocalization of macrophages in air pouch from Balb/c mice after 4- and 8-hour inoculation. (a) Number of macrophages per field. Groups (*n* = 6): Tween 1% (C), citral (CT),* S. aureus* (S), and* S. aureus* treated with citral (S + CT). 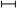
Significant difference with *p* < 0.05, Kruskal-Wallis test. (b) Representative photomicrographs of immunolocalization of macrophages. Original magnification ×400.

The expression of antibody CD 68 was seen in the brownish color marking of macrophages, being evaluated by immunohistochemistry. There was a response variation between the groups of infected mice compared to the infected and treated group with the EOC, or the group that received the EOC, the time of 4 h, with significant differences, *p* < 0.05 (Figure 6).

## 4. Discussion

In infections, an appropriate response is an important aspect that will distinguish whether the host will be cured or not. The use of biologically active natural products has been increasing in recent decades, becoming an alternative for treating various infections. However, scientific evidences on effectiveness and mechanisms of action for these derivatives are limited, hence, delaying their use in clinical practice [19]. The air pouch tissue is composed of different cell lineages, macrophages, and fibroblasts initially, similar to a synovial cavity, allowing analysis of the inflammatory response, similar to human joint inflammation in rheumatoid arthritis [20].

In the present study, analyzing bacterial load, it was observed that the EOC had a positive effect, since the load of* S. aureus* was reduced in the inflammatory environment when treated on both study days. However, few data report the action of EOC on microorganisms in a model air pouch. Studies assessing ginger oils, lemongrass (has high citral content), mint, pepper, and rosemary against a strain of* S. aureus* have reported bactericidal effects of these oils after 24 h [21]. It was observed that the EOC does not induce the development of resistance to antibiotics or components of* S. aureus* and that subinhibitory concentrations (0.05 mg mL^−1^) significantly inhibit the formation of biofilm, being considered an important antimicrobial agent [22]. It is believed that the essential oils target different molecular targets for antibiotics, being active against pathogens often resistant to conventional therapy [20]. Other studies report that some essential oils may contain substances that more easily penetrate the lipid layer, as differences in the bacterial cell wall structures allow or prevent entry of substances in bacteria [23].

During inflammatory processes, some mononuclear cells and polymorphonuclear immune system are recruited to the site of infection and can initiate an inflammatory response [24]. Regarding the profile of total leukocytes in peripheral blood, we found no differences between the S and S + CT groups. However, increases were observed in the number of cells in C and CT groups. This increase can be influenced by many factors and cannot be solely related to bacterial action, since the air pouch induction causes stress in the animals with consequent release of catecholamines and cortisol, which may lead to an increase in the blood rate of leucocytes [25]. The differential counts of leukocytes (lymphocytes, neutrophils, and monocytes) showed differences in the two times of the experiment. Although there were no differences observed between the groups S and S + CT compared to neutrophils, they are among the major cell types elevated in the bloodstream after a bacterial infection, part of the first line of defense [26]. Different studies with* S. aureus* demonstrate an increase of neutrophils in the peripheral blood after challenge [27, 28], unlike the data found in the two experiments in this study. Two receptors are involved in the assembly of the inflammatory response and migration of neutrophils to the site of inflammation, NOD2 and TLR2 [29]. In the present study, groups C, CT, and S + CT had a higher migration of neutrophils compared to group S. When infection occurs, several chemoattractant factors are released and can be recognized by receptors on the surface of neutrophils, resulting in a strong chemoattractant response that can stimulate and activate a cascade of intracellular signaling, providing neutrophil migration to the inflammatory site [30].

The air pouch model, the local reaction at the infection site, has as its trigger a localized inflammatory response, wherein the release of cytokines can occur so that they eradicate infection [28]. In this study, the analysis of the washed air pouch was observed for all three cytokines studied, showing a reduction in the levels when the animals were treated with EOC. The anti-inflammatory activity of this oil was described [31], demonstrating that it has the property of inhibiting production of in vivo and in vitro IL-1*β* and IL-6 in mice peritoneal macrophages, as confirmed by findings in this study in relation to IL-6. According to the literature, mice inoculated with carrageenan, the treatment with oral doses of 50–300 mg/Kg of essential oil obtained from* Myrcia ovata* plant, rich in EOC, demonstrated a significant effect in reducing pain and inflammation [23]. Regarding the data obtained in the washing with the inflammatory mediators of the air pouch tissue, this result may have occurred due to the mechanism of action, since the pharmacological effect of the most relevant EOC is caused by inhibition of TRPV1, TRPV3, TRPA1, and TRPM8 after activation of these receptors. This inhibition occurs only in initially activated receptors and is irreversible and calcium-independent [32].

Analyzing the expression of TNF-*α*, IL-1*β*, and IL-6 gene, we can observe significant differences for the three genes. However, evaluating the S and S + T groups, only in the time of 4 h there were no significant difference levels of TNF-*α*. Thus, it is believed that the EOC has anti-inflammatory abilities, since it enhances TNF-*α* as a target for the prevention of inflammatory events induced by chemicals. Although few studies can be found in the literature on the influence of monoterpene compounds in modulating gene expression of TNF-*α*, there are reports that citral oil inhibits an increase in TNF-*α* levels in RAW 264.7 cells that are stimulated with lipopolysaccharide (LPS) [33]. In the same way, it was observed in the literature that the EOC at concentrations of 50 and 100 *μ*g/mL reduced the TNF-*α* relative expression compared to treatment with LPS [34]. Authors analyzing alveolar macrophages observed that treatment with citral oil in mice with lung injury induced by LPS inhibited TNF-*α*, IL-1*β*, and IL-6 levels both in vivo and in vitro, demonstrating that the EOC can inhibit a possible inflammatory response [18]. It was also demonstrated that the alcoholic extract of lemongrass, which has as major compound citral, reduced the generation of TNF-*α* in bronchoalveolar macrophages stimulated with LPS, enhancing the anti-inflammatory potential of citral and indicating that modulation of the COX-2 and TNF-*α* genes can be one of the mechanisms involved in such activity [35].

The dosage of inflammatory markers by ELISA compared to gene expression showed differences in results, particularly IL-6 and IL-1*β*. This could happen through a change in the amount of cytokines, due to the expression or not of a gene regulated by a site-specific methylation. The immunological response may be compromised by epigenetic mechanisms, including gene silencing by methylation in the promoter region and blocking gene transcription [36]. In addition, the EOC may cause possible interference in the expression of these genes. In the line RAW 264.7 mice macrophages stimulated with LPS, citral inhibited the phosphorylation interaction with inhibitory proteins kB (IkB), blocking translocation of the p50 and p65 subunits of NF-kB and leading to a low expression of inducing enzyme nitric oxide synthetase (iNOS) [37].

This study also demonstrated a relationship between the production of cytokines and bacterial load, since the lower bacterial load in animals treated seems to be related to lower IL-6, IL-1*β*, and TNF-*α* expression in washed air pouch and TNF-*α* of gene expression. Regarding the effectiveness in times of treatment chosen, it seems that with 4 h the EOC had better action; however, little is known about the performance of this oil in animals, and more studies need to be conducted. A study on the stability of the major constituents of the essential oil of lemongrass, rich mainly in citral and myrcene, reported a significant variation in the degradation of these compounds in relation to temperature and time; however, the interaction between the variants was not significant for citral [38].

Thus, our results showed that the use of the essential oil citral in the local therapy of infectious diseases triggered by* S. aureus* showed positive action. However, the existence of a harmful action or interference in the physiology and/or structures of bacterial cells of this natural product and its bioavailability when used in living beings are not well understood. Given the above information and grounded in the literature, this was the first study to assess the joint influence of the air pouch model, essential oil citral, and* S. aureus*, since the EOC appears to be a potential therapeutic agent for treating local infections triggered by* S. aureus*. In conclusion, the treatment with essential oil citral in the infection triggered by* S. aureus* led to a reduction of some features of acute inflammation, including the number of monocytes. The TNF-*α* cytokine has proved to be a more sensitive biomarker, in ELISA and RT-qPCR array. By reducing TNF-*α* concentration, EOC promoted the reduction of transcription of genes related to proinflammatory cytokines. The action of the EOC seems to have a better response in a period of 4 h; thus, this suggests that the EOC can act as a modulator of the immune system by decreasing cellular migration and the production of proinflammatory cytokines following infection with* S. aureus*.
